# Fluctuating thermal environments and time‐dependent effects on fruit fly egg‐hatching performance

**DOI:** 10.1002/ece3.4220

**Published:** 2018-06-21

**Authors:** Grisel Cavieres, José M. Bogdanovich, Paloma Toledo, Francisco Bozinovic

**Affiliations:** ^1^ Departamento de Ecología Center of Applied Ecology and Sustainability (CAPES‐UC) Facultad de Ciencias Biológicas Pontificia Universidad Católica de Chile Santiago Chile; ^2^ CCT‐Mendoza CONICET Grupo de Investigaciones de la Biodiversidad CONICET Instituto Argentino de Investigaciones de Zonas Áridas Mendoza Argentina; ^3^ Centro de Investigación e Innovación para el Cambio Climático Universidad Santo Tomás Santiago Chile

**Keywords:** *Drosophila melanogaster*, hatching success, thermal variability, time‐dependent effects

## Abstract

Organismal performance in a changing environment is dependent on temporal patterns and duration of exposure to thermal variability. We experimentally assessed the time‐dependent effects of thermal variability (i.e., patterns of thermal exposure) on the hatching performance of *Drosophila melanogaster*. Flies were collected in central Chile and maintained for four generations in laboratory conditions. Fourth generation eggs were acclimated to different thermal fluctuation cycles until hatching occurred. Our results show that the frequency of extreme thermal events has a significant effect on hatching success. Eggs exposed to 24 hr cycles of thermal fluctuation had a higher proportion of eggs that hatched than those acclimated to shorter (6 and 12 hr) and longer cycles (48 hr). Furthermore, eggs subjected to frequent thermal fluctuations hatched earlier than those acclimated to less frequent thermal fluctuations. Overall, we show that, egg‐to‐adult viability is dependent on the pattern of thermal fluctuations experienced during ontogeny; thus, the pattern of thermal fluctuation experienced by flies has a significant and until now unappreciated impact on fitness.

## 1 INTRODUCTION

Insights about the suitability of a thermal landscape for a given species should consider not only average thermal values but also variability in thermal values (Bozinovic, Medina, Alruiz, Cavieres, & Sabat, 2016; Bozinovic, Sabat, Rezende, & Canals, 2016; Estay, Lima, & Bozinovic, 2014). Theoretical (Katz, Brush, & Parlange, 2005) and empirical studies (Easterling et al., 2000) have demonstrated that daily and seasonal variation in temperature affects organisms’ ecology and fitness (Clavijo‐Baquet et al., 2014; Gilbert & Miles, 2017; Kielland, Bech, & Einum, 2017; Messenger & Flitters, 1958; Roitberg & Mangel, 2016; Saarinen, Laakso, Lindström, & Ketola, 2018). In addition, thermal extremes, defined as events that alter the distribution of ambient temperature without influencing the mean and the variance (Ummenhofer & Meehl, 2017), have also been shown to have outstanding effects on physiological performance and survival (Bozinovic, Medina, et al., 2016; Kingsolver & Woods, 2016).

Experimental studies have evaluated the effect of thermal variability on physiological and life‐history traits and have shown that organisms can respond to thermal variability through plasticity, and such responses could affect current and future fitness (Bozinovic, Catalan, Estay, & Sabat, 2013; Chevin & Hoffmann, 2017; Colinet, Sinclair, Vernon, & Renault, 2015; Fischer, Klockmann, & Reim, 2014; Manenti, Loeschcke, Moghadam, & Sørensen, 2015; Meats, 2011; Terblanche, Nyamukondiwa, & Kleynhans, 2010). Further to this, multiple studies suggest that the ability to cope with thermal fluctuations rather than just tolerate different mean temperatures is probably of much greater importance to species survival and thermal adaptation (Boher, Trefault, Estay, & Bozinovic, 2016; Dey, Proulx, & Teotónio, 2015; Kubrak, Nylin, Flatt, Nässel, & Leimar, 2017; Marshall & Sinclair, 2010). Environmental thermal variability in space and time imposes selective pressures on organisms (Bozinovic, Medina, et al., 2016; Clavijo‐Baquet et al., 2014; Gould, 1985; Levins, 1968), and performance can be affected by increased variability in temperature even if the mean temperature does not change (Bozinovic, Medina, et al., 2016; Cavieres, Bogdanovich, & Bozinovic, 2016; Vázquez, Gianoli, Morris, & Bozinovic, 2017). Nevertheless, little attention has been given to quantifying the effects of the duration and patterns of thermal exposure on ectotherm performance and fitness (time‐dependent effects sensu Kingsolver & Woods, 2016), but see Roitberg and Mangel (2016). According to Kingsolver and Woods (2016), theoretical and empirical studies of time‐dependent effects are the necessary first steps for generating explanations and building strong predictions about the consequences of climatic change (Bozinovic & Pörtner, 2015; Koussoroplis, Pincebourde, & Wacker, 2017).

Overall, tolerance to extreme temperatures has been shown to differ depending on temporal scale of exposure (Nguyen, Bahar, Baker, & Andrew, 2014). For instance, Chidawanyika and Terblanche (2011) report increases of 70% in the survival of moths exposed to high temperatures for short periods of time (37°C/1 hr) (see also Khani & Moharramipour, 2009; Overgaard & Sørensen, 2008; Rozsypal, Koštál, Zahradníčková, & Šimek, 2013; Sinclair, Jaco Klok, Scott, Terblanche, & Chown, 2003). In addition, the response of organisms to thermal fluctuation can differ depending on the nature of thermal fluctuation. For example, Ketola, Kellermann, Loeschcke, López‐Sepulcre, and Kristensen (2014) do not report significant effects of exposure to thermal fluctuations (with the mean temperature remaining constant) on egg‐to‐adult viability in *Drosophila melanogaster*. Nevertheless, Bozinovic, Medina, et al. (2016) tested the effect of thermal change on fly survival and found 85% reductions in the survival of adult flies acclimated to thermal fluctuation involving changes in temperature variance. Also, in that study, no significant differences were found in the survival of fruit flies acclimated to thermal variability but with constant mean temperatures.

Given that thermal conditions impact individual performance, plasticity, and survival, here we experimentally assess how thermal fluctuation (i.e., thermal cycle duration) affects life‐history and ultimately fitness to better understand how organisms cope with rapid environmental changes. We used the fruit fly *D. melanogaster* from central Chile as an ectothermic model (see Methods). Earlier, this species has been used to test hypotheses about the impacts of thermal variability on performance and fitness (Bozinovic et al., 2013). Furthermore, the phenotypic responses of this species to environmental temperature and other climatic factors are well known (Castañeda, Balanyà, Rezende, & Santos, 2013; Castañeda, Rezende, & Santos, 2015; Hoffmann, 2010; Ohtsu, Katagiri, Kimura, & Hori, 1993; Parkash, Aggarwal, Singh, Lambhod, & Ranga, 2013; Ragland & Kingsolver, 2008). Thus, here, we determine the time‐dependent effects of thermal variation on fruit fly hatching performance. We hypothesized that the effect of thermal variability would depend on the frequency of exposure to extreme temperatures. In addition, we postulated that hatching success would decline with increasing frequency of exposure to extreme temperatures. Overall, it was thought that repeated suboptimal temperature shocks would induce stress in our animal model, and thus, fitness would decrease with increased exposure.

## 2 METHODS

Adult *D. melanogaster* flies were collected in central Chile (33°26′S; 70°39′W at 500 m above sea level) during summer 2016. After capture, flies were identified based on morphological characters (Markow & O'Grady, 2005) and from these, ten breeding groups were generated. Each group consisted of proximately 10 males and 10 females. Flies were reared in controlled conditions (24°C and LD = 12:12) in 250 ml glass vials with Burdick culture medium (Burdick, 1955). The groups were maintained for three generations. Third generation virgin flies were collected within 8 hr of hatching and were transferred to vials containing 11 g of culture medium. Two individuals (sex ratio 1:1) were maintained in each vial. After 24 hr, 20 eggs were collected from each vial using a microscope. These eggs were transferred in batch to fresh vials. Vials, each containing twenty eggs, were assigned to either the control group (28 ± 0°C) or to one of four thermal treatments that differed in the frequency pattern of thermal fluctuation (28 ± 4°C). The frequency pattern of thermal fluctuation for each treatment was composed of cycles of (a) 6 hr (*n* = 13 replicates); (b) 12 hr (*n* = 10 replicates); (c) 24 hr (*n* = 10 replicates); and (d) 48 hr (*n* = 12 replicates). In all treatments, temperature increased linearly, reached the maximum temperature (32°C), remained constant, and then began to decrease until a minimum temperature (24°C) was reached. All cycles were repeated until egg‐hatching was completed. The photoperiod for each treatment was LD = 12:12. The heating/cooling rate between the minimum and maximum temperatures was 0.26°C/ min, allowing them to spend most of the treatment time in the maximum and minimum temperatures. The thermal fluctuation range (28 ± 4°C) was set based on the well‐known limits of fruit fly egg viability (Hoffmann, 2010). The proportions of hatching eggs in the upper (32 ± 0°C, *n* = 14 replicates) and lower (24 ± 0°C, *n* = 14 replicates) thermal fluctuation treatments were 0% and 34%, respectively. Eggs were maintained in their respective treatments until hatching occurred. Later, hatching performance was evaluated as the proportion of flies found in the replicate vials every 24 hr (i.e., accumulative hatching success within 24 hr) during a total period of 12 days; we quantified the amount of adults that successfully developed, yet we did not check for stages of development (e.g., larvae and pupae). In particular, we quantified (a) proportion of hatched eggs (total) and (b) hatching success over time. Normality and homoscedasticity assumptions were fulfilled using square root and exponential function transformations.

The proportion of hatched eggs was analyzed using a general linear model with a negative Binomial distribution. To analyze hatching success over time, we tested the effect of treatment and time until hatching (days) on the proportion of hatched eggs; the data were fitted to a second‐order fractional polynomial function. Then, a linear mixed model was generated for the longitudinal data; number of replicates (random intercept) was nested in time until hatching (slope) and was included as a random effect. Differences among treatments were tested using post hoc comparisons (Tukey tests). The statistical analyses were carried out in R (R Core Team, 2017). The datasets analyzed during the current study are available in the Dryad Digital Repository.

## 3 RESULTS

**Figure 1:**
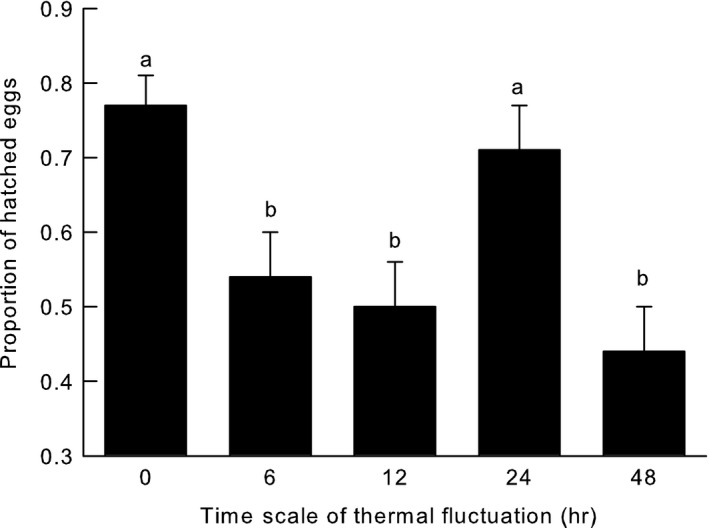
Proportion of hatched *Drosophila melanogaster* eggs acclimated to different timescales of thermal fluctuation (6, 12, 24, 48 hr, and control [0 hr]). Different letters indicate significant differences between values. Data are reported as mean ±*SE*

Egg viability differed significantly among treatments (Figure 1). In particular, the proportion of eggs that hatched was lower for flies acclimated to long (48 hr) and short (6 and 12 hr) thermal fluctuation timescales compared to flies subjected to control (0 hr) and moderate (24 hr) timescales of thermal variability.

**Figure 2:**
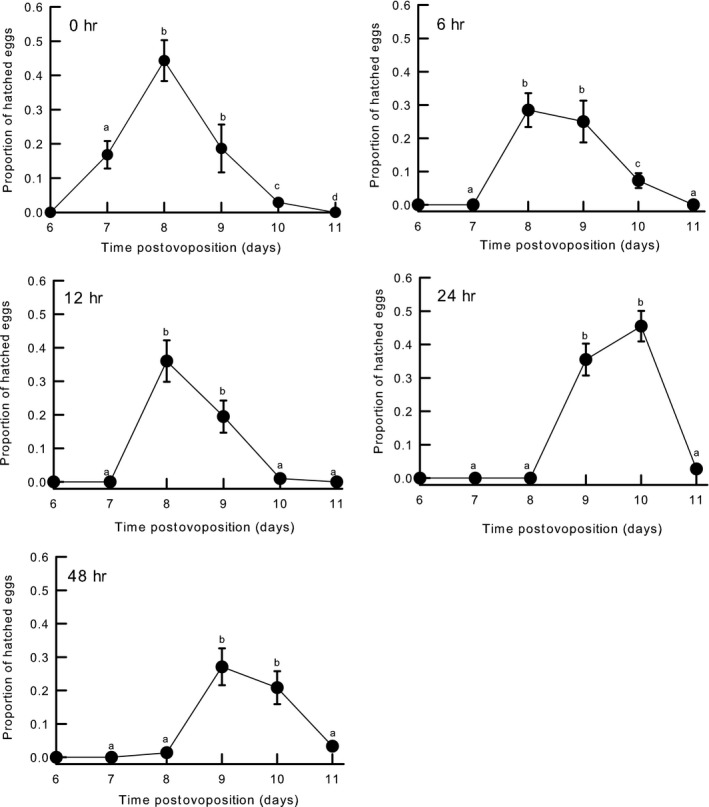
Time that passed until *Drosophila melanogaster* eggs hatched. Eggs were acclimated to different frequency of thermal fluctuation (6, 12, 24, 48 hr, and control [0 hr]). Different letters indicate significant differences between values. Data are reported as mean ±*SE*

Compared to the control group, where 77% of eggs hatched, the proportions of eggs hatching in the 6, 12, and 48 hr treatments were 54%, 50%, and 48% (Figure 1, Table 1). In addition, hatching success over time differed significantly among treatments (Table 1, Figure 2); eggs in the 24 and 48 hr treatments hatched later than eggs in the control, 6, and 12 hr groups (Figure 2).

**Table 1:** Coefficients of the linear and fractional polynomial models fitted to hatching performance of *Drosophila melanogaster*. Eggs were maintained in one of four treatments that differed in timescales of thermal variability (6, 12, 24, 48 hr, and control)

Effect	Estimate	*SE*	*T*	*p*
Proportion of hatched eggs				
Intercept	0.77	0.04	16.82	**<0.001**
6 hr	−0.23	0.06	−3.73	**<0.001**
12 hr	−0.27	0.06	−4.06	**<0.001**
24 hr	0.06	0.06	1.01	0.77
48 hr	−0.33	0.06	−5.14	**<0.001**
Hatching success over time				
Intercept	−5.32	0.59	−9.01	**<0.001**
Intercept (Control)	−9.55	1.03	−9.2	**<0.001**
6 hr	−1.61	0.51	−3.12	**0.002**
12 hr	−1.21	0.55	−2.21	**0.035**
24 hr	−3.77	0.55	−6.86	**<0.001**
Time	6.21	0.60	10.31	**<0.001**
Time^2^	−0.04	0.003	−12.67	**<0.001**
6 hr × Time	0.71	0.23	3.08	**0.002**
12 hr × Time	0.52	0.24	2.12	**0.035**
24 hr × Time	1.72	0.24	6.98	**<0.001**
48 hr × Time	1.57	0.23	6.67	**<0.001**

## 4 DISCUSSION

Adaptation to varying thermal environments depends on the temporal pattern of environmental changes and the tolerance of each phenotype (Levins, 1968). Thus, environmental variability in time and space imposes challenges on organisms (Gould, 1985) where animals that inhabit variable environments are expected to be plastic in order to survive a broad range of temperatures.

Some studies have evaluated the effect of thermal variability on life‐history traits, including developmental time (Ragland & Kingsolver, 2008), hatching success (Ji, Gao, & Han, 2007), and phenotypic characteristics of progeny (Estay, Clavijo‐Baquet, Lima, & Bozinovic, 2011; Folguera, Bastías, & Bozinovic, 2009; Krams, Daukšte, Kivleniece, Krama, & Rantala, 2011; Orcutt & Porter, 1983; Paaijmans et al., 2010; Pétavy, David, Debat, Gibert, & Moreteau, 2004; Williams et al., 2012). In nature, ectotherms must continuously cope with short‐ and long‐term environmental variation (Buckley & Huey, 2016; Grant et al., 2017; Kingsolver & Buckley, 2017; Wingfield et al., 2017), and some organisms perform well and are highly tolerant to variable conditions (Angilletta, Wilson, Navas, & James, 2003; Condon, Cooper, Yeaman, & Angilletta, 2014; Seebacher, Ducret, Little, & Adriaenssens, 2015). On the contrary, other species deal with variability through thermal acclimation and/or acclimatization (Terblanche et al., 2010). Knowing how fluctuating thermal conditions affect individual performance and plastic responses is central for predicting organismal responses to climatic change (Kingsolver & Buckley, 2017; Koussoroplis et al., 2017).

Our key finding here was that frequency of thermal stress experienced by fly eggs affects hatching performance. Some studies that have assessed the impact of thermal fluctuations on egg‐to‐adult viability have shown contrasting results (Manenti et al., 2015; Masel & Siegal, 2009), and most other studies have only considered intermediate scales of thermal fluctuation (e.g., 24 hr). Here, we observed that hatching performance was higher in flies maintained in thermal treatments for 24 hr compared to the hatching performance of flies maintained in treatments for 6, 12, and 48 hr (Figure 2). In addition, eggs maintained in shorter cycles of thermal fluctuation (6 hr) hatched earlier than those that experienced longer cycles. As expected, repeated heat shocking negatively affected the performance and fitness of our experimental flies. According to Ketola et al. (2014), the high performance of *D. melanogaster* in variable environments is explained by high average viability across environments and not by having high environmental robustness in terms of viability (i.e., low variation in viability across environments) (Ketola et al., 2014; Liefting, Hoffmann, & Ellers, 2009; Masel & Siegal, 2009). In theory, when eggs are exposed to a high frequency of stressful temperatures (32°C; 6 and 12 hr treatments), reductions in hatching performance are probably a consequence of the increased energetic costs of maintenance and synthesis of heat‐shock proteins (HSPs; Chevin & Hoffmann, 2017; Cooper Brandon, Hammad Loubna, Montooth Kristi, & Robbie, 2014; Kafri, Metzl‐Raz, Jona, & Barkai, 2016; Podrabsky & Somero, 2004); however, these costs were not assessed in this study. For instance, Krebs and Feder (1997) report that HSPs are biosynthesized and degraded nearly every 1.5 and 4–6 hr, respectively (see also Sørensen, Nielsen, Kruhøffer, Justesen, & Loeschcke, 2005; Tomanek & Somero, 2000). Thus, frequent events of high temperatures that cause physiological stress may cause organisms to have to reduce energy allocations to long‐term processes such as those involved in reproduction and hatching; hence, fitness could be negatively impacted. Also, increased ambient temperature could affect the circadian rhythms of hatching and locomotion in fruit flies (Rosbash et al., 1996). A similar hypothesis could be stated for longer times of exposure to high and stressful temperatures such as that experienced by flies in the 48 hr treatment. Indeed, according to Roberts and Feder (2000) prolonged duration of thermal exposure has negative effects on performance and fitness; specifically, chronic exposure to high temperatures reduces insect survival and rate of development (Kingsolver & Woods, 2016; Krebs & Feder, 1997).

Here, we have shown that thermal fluctuation significantly impacts hatching success. Despite this, consequences to fitness may not only depend on the direct effects of extreme temperatures (Foray, Desouhant, & Gibert, 2014; Kostál, Renault, Mehrabianová, & Bastl, 2007), but environmental conditions experienced by parents could affect performance and fitness in their offspring (Salinas et al., 2013). Thus, transgenerational effects can mediate the negative impacts of extreme events (Donelson, Salinas, L. Munday, & Shama, 2017; Donelson, Wong, Booth, & Munday, 2016; Mousseau & Dingle, 1991; Rodríguez‐Romero, Jarrold, Massamba‐N'Siala, Spicer, & Calosi, 2016). As a consequence, future studies that incorporate the thermal history of organisms are important to predict the probable evolution and/or acclimation capacities of thermal sensitivity in nature.

Summarizing, variable and extreme environments are characterized by the increased frequency and harshness of thermal events. Nevertheless, the definition of extreme environments in a biological context needs to consider the biological response of organisms to such extreme events. As suggested by Chevin and Hoffmann (2017), the definition of fluctuating and extreme environments is organism‐specific, because a stressful environment to one organism or species may not be stressful or could even be benign to another species. At last, our results support the hypothesis that hatching performance is dependent on patterns of thermal fluctuation experienced throughout ontogeny. This fact highlights the importance of incorporating thermal fluctuation in studies of phenotypic variation. Indeed, as demonstrated here, an understanding of the effect of the patterns of thermal exposure on fly fitness is necessary for building predictions about the consequences of climatic variability (Bozinovic, Sabat, et al., 2016; Kingsolver & Woods, 2016). Although controlled experiments cannot capture the widespread range of thermal environments in nature, simple tests such as those presented here can shed light on the mechanisms involved in responses to thermal variability. Moreover, these types of experiments can shed light on the causes of phenotypic variation at different temporal and spatial scales as environmental temperature varies over time as well as across geographic gradients.

## CONFLICT OF INTEREST

None declared.

## AUTHOR CONTRIBUTIONS

G.C., J.M.B. and F.B. designed the experiment. G.C. and P.T. performed the experiments. G.C. and J.M.B. analyzed the data. G.C. and F.B. wrote the paper.

## DATA AVAILABILITY

Data are available at the Dryad Digital Repository.
